# Assessment of the Implementation of Combined Physical Activity and Nutrition Programmes in Schools: A Systematic Review

**DOI:** 10.3390/healthcare14132029

**Published:** 2026-07-07

**Authors:** Rafael Francisco Caracuel-Cáliz, Francisco Rivas García, José Manuel Armada-Crespo, Manuel Tomás Abad Robles

**Affiliations:** 1 Faculty of Education Sciences, Valencian International University, 46002 Valencia, Spain; rafaelfrancisco.caracuel@professor.universidadviu.com; 2 Faculty of Education and Humanities, International University of La Rioja, 26006 Logroño, Spain; 3 Guadix City Council, 18500 Guadix, Spain; saludyconsumo@guadix.es; 4 School of Health Sciences, Valencia International University, 46002 Valencia, Spain; 5 Research Group in Sport and Physical Education for Personal and Social Development (GIDEPSO), Department of Specific Didactics, University of Cordoba, 14071 Córdoba, Spain; 6 Department of Integrated Didactics, University of Huelva, 21007 Huelva, Spain; manuel.abad@dempc.uhu.es

**Keywords:** school-based interventions, physical activity, nutrition education, health promotion, children, adolescents, implementation, school health, health literacy, obesity prevention

## Abstract

Background/Objectives: The rise in unhealthy habits such as a sedentary lifestyle, coupled with increasing obesity rates among children and young people, presents a health problem that must be addressed from various perspectives. Thus, the educational context, and specifically physical education, offers a prime setting for implementing programmes that reduce the levels of physical inactivity and improve pupils’ nutritional behaviours. Therefore, the aim of this study was to conduct a systematic review to collect and analyse the scientific literature addressing the effects of school-based intervention programmes on physical activity and nutrition. Methods: To this end, a systematic review was carried out, based on the PRISMA method, in the ERIC, PubMed, Scopus, SportDiscus and Web of Science databases, analysing the scientific literature that included school-based interventions combining physical activity and nutrition. A total of 410 articles were identified, with 16 studies ultimately included following the application of the inclusion and exclusion criteria. Results: The results indicated that the implementation of school programmes combining physical activity and nutrition can bring benefits in both areas, or at least in one of them. Similarly, several of the studies analysed showed improvements within the community, which helped to increase health literacy. Conclusions: The main conclusion is that the educational setting can serve as a platform for implementing programmes that improve the physical activity and nutritional habits of pupils and their wider environment, and, crucially, enhance health literacy.

## 1. Introduction

Currently, there is an increase in sedentary lifestyles and unhealthy habits among young people, as indicated by the World Health Organization (WHO) [1]. In this regard, the educational context in general, and physical education (PE) in particular, offers favourable conditions for addressing health-related issues through programmes that combine nutrition and physical activity (PA) [2,3,4].

The educational context can be one of the key elements for promoting physical and nutritional health through programmes that combine these aspects, but establishing a link with the community and educational policies is essential for this to have real and sustainable effects [2,5]. Given the possibility of incorporating health-based strategies into PE, due to its curricular nature, many countries have chosen to integrate physical and health literacy into the subject itself as a further context for intervention within comprehensive PA programmes [6]. With regard to educational policies, a recent study confirms the value of incorporating lessons on nutrition and PA into the educational curriculum, alongside other complementary activities, to increase health literacy and improve anthropometric habits and values [7].

Furthermore, with regard to the implementation of PA and nutrition programmes, there are a number of key factors that determine whether the programme developed is capable of bringing about improvements in participants and whether these improvements can be attributed to the programme [8]. These key factors are adherence, acceptability, and the barriers and facilitators to the implementation of the intervention.

For instance, several school-based nutrition and PA programmes have evaluated their interventions not only for overall effectiveness but also in terms of implementation fidelity, acceptability, barriers, and facilitators [9,10,11].

These concepts can be considered the foundations of the sustainability of interventions. Regarding the feasibility of implementing these programmes in schools, the presence of school administrators who are sensitive to these issues and of teaching staff with the necessary skills and motivation to promote interventions based on nutrition and PA has a positive impact on their sustainability [12]. In this regard, in addition to the programme’s own characteristics, teachers’ competencies are fundamental to its implementation. Thus, a study conducted with 203 schoolchildren, in which an intervention proposal based on team pentathlon was implemented, revealed that the teacher themselves and their methodology, together with the resources employed, were the determining factors for a significant increase in PA among the pupils [13].

In line with the sustainability of interventions, a recent systematic review addressing barriers and facilitators in the implementation of PA and nutrition programmes for adults concluded that social support received from the context is key to the success of the intervention [8]. This is also observed in a study in an educational setting involving 1630 primary school pupils in China, in which a 12-month programme involving families and pupils was implemented, concluding that following the programme, the pupils had improved in terms of weight, dietary intake and higher levels of PA compared to the control group [14].

Thus, and in terms of programme effectiveness, school-based PA interventions tend to have a greater impact in longer-term programmes [15], whilst the effectiveness of school-based nutrition interventions should be directed towards a multi-component approach [16]. The existing literature on this topic focuses on the effectiveness of nutrition or PA programmes [15], but there are little data on their integration through programmes in an educational context, despite the fact that recent publications have called for this [17] and others have highlighted the value of a multi-component approach [18]. The aim of this study was therefore to conduct a systematic review to compile, analyse and describe the scientific literature addressing the effects of school-based intervention programmes on PA and nutrition.

## 2. Materials and Methods

A systematic review was conducted in accordance with the PRISMA guidelines [19] (Supplementary Material S1) for the identification, selection and synthesis of the included studies. Furthermore, the PICO strategy [20] was used to define the eligibility criteria, and the methodological recommendations for systematic reviews proposed by Moher et al. [21] were taken into account. Studies conducted between 2020 and 2026 were analysed.

The review methods were defined prior to the start of the study and registered in the International Prospective Register of Systematic Reviews (PROSPERO) under identification number CRD420261394552, available at https://www.crd.york.ac.uk/PROSPERO/view/CRD420261394552 (accessed on 17 May 2026).

### 2.1. Eligibility Criteria

The inclusion criteria were as follows:

(a) Availability of the full text.
(b) Original articles (excluding systematic reviews, meta-analyses and protocols).
(c) Written in English, Spanish or Portuguese.
(d) Studies conducted in school-age populations (5–18 years) that included educational interventions combining nutrition education and PA.
(e) Studies evaluating outcomes related to implementation, effectiveness, or both.
(f) Experimental, quasi-experimental or implementation studies (including qualitative or mixed-methods approaches when addressing implementation processes).

The exclusion criteria were based on the absence or omission in the reviewed articles of the inclusion criteria listed above. Consequently, studies that addressed only PA or only nutrition without a combined component, observational studies without an intervention, and research that did not assess variables related to the implementation or effects of the intervention were excluded. The application of these criteria ensured the selection of studies aligned with the objective of analysing multi-component school-based interventions and their implementation.

Studies that did not meet the eligibility criteria defined for the review were excluded. Specifically, systematic reviews, meta-analyses, scoping reviews or corrections, such as Navidad et al. (2021) [15]; protocols without empirical results, such as [22]; studies conducted outside the school context, such as Ashton et al. (2024) [23]; research involving university students or adults, such as Al-Qahtani et al. (2022) [24] or Hoosen et al. (2024) [25]; observational studies without intervention, such as Pérez-Mármol et al. (2021) [26]; interventions focused exclusively on nutrition, such as Michael & Talias (2024) [27], or exclusively on PA, such as [28]; qualitative studies not linked to a specific intervention, such as Mohammadi et al. (2021) [29]; methodological or planning studies without implementation results, such as O’Byrne et al. (2024) [30]; specific clinical protocols or interventions, such as Zhang et al. (2024) [31]; and programmes developed mainly in out-of-school or non-curricular contexts, such as Weaver et al. (2024) [32] (Supplementary Material S2).

In order to present information that met the quality standards of high-impact journals, all the documents reviewed were drawn from the scientific literature, excluding the grey literature or non-indexed publications.

### 2.2. Search Strategy

The literature search was conducted in accordance with the PRISMA guidelines [19]. To this end, a search strategy was designed based on a combination of terms related to school-based interventions, nutrition, PA and implementation. The search query used was as follows:

(“primary education” OR “primary school” OR “elementary school” OR “basic education” OR “secondary school” OR “compulsory secondary education” OR “obligatory secondary education” OR “secondary education”) AND (school* OR “school-based” OR “educational setting*” OR classroom* OR “physical education” OR “after-school” OR “school programme*”) AND ((“physical activity” OR exercise OR sport* OR “physical education”) AND (nutrition OR diet* OR “nutrition education” OR “healthy eating”)) AND ((intervention OR experimental OR quasi-experimental OR “randomised controlled trial” OR RCT OR “controlled trial”) AND (implementation OR “implementation science” OR “programme implementation” OR fidelity OR adherence OR “process evaluation” OR “implementation fidelity” OR barrier* OR facilitator* OR acceptability OR feasibility OR “reach” OR “dose delivered” OR “dose received” OR adoption OR “RE-AIM” OR “CFIR” OR “implementation framework*” OR “mixed methods”)).

Searches were conducted in the ERIC, PubMed, Scopus, SportDiscus and Web of Science databases during the period from 6 November 2025 to 27 February 2026. In addition, filters were applied based on publication type, date range, and languages. The search strategy was adjusted to suit each database.

Following the retrieval of records, duplicates were removed and an initial screening of the results was carried out.

### 2.3. Study Selection and Data Processing

In the first phase, titles and abstracts were reviewed to identify potentially relevant studies [33]. Subsequently, the full texts of the selected articles were read to verify compliance with the inclusion criteria.

**Figure 1:**
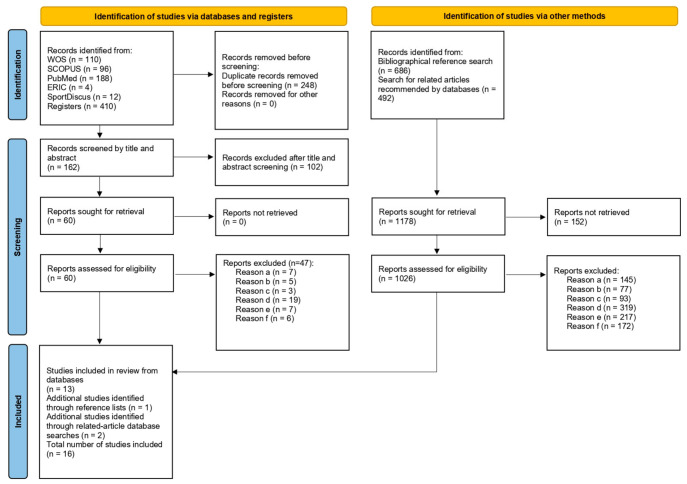
Flow diagram (PRISMA, 2020) (Supplementary Material S3).

The selection process was carried out independently by two researchers. In the event of discrepancies, these were resolved by consensus or, where necessary, with the intervention of a third researcher, ensuring the objectivity of the process [34]. The study selection flow is presented in Figure 1.

### 2.4. Methodological Quality and Risk-of-Bias Assessment

The methodological quality of the included studies was assessed using the Standard Quality Assessment Criteria tool [35]. This tool was retained because the review included studies with different methodological designs, including quantitative, quasi-experimental, mixed-methods, protocol/design and implementation-focused studies. Scores were calculated as proportions of the maximum applicable score. Two researchers independently analysed each study, considering aspects such as design, sample, methods, analysis and presentation of results.

The items were scored according to the degree of compliance. Thus, 2 points were awarded where the criterion was fully met, 1 point where compliance was partial, and 0 points where it was not met. Where the criterion was not applicable, NA’ was indicated. The final score for the quantitative studies was calculated using the formula: [(‘satisfactory items’ × 2) + (‘partially satisfactory items’ × 1)/28 − (‘not applicable items’ × 2)]. The results for the qualitative studies were obtained using the formula: [(‘satisfactory items’ × 2) + (‘unsatisfactory items’ × 1)/20]. The results were expressed on a scale from 0 to 1.

The level of agreement between assessors was determined using the intraclass correlation coefficient, yielding a value of 0.876 (*p* < 0.001), which indicated a good level of agreement [36]. A minimum cut-off point of 65% was established for the inclusion of studies in the final analysis with the aim of setting a conservative standard regarding the quality of the included studies.

In addition, a complementary domain-based risk-of-bias assessment was conducted according to study design. Randomised controlled trials and cluster-randomised controlled trials were assessed using the revised Cochrane risk-of-bias tool for randomised trials, RoB 2 [37]. For cluster-randomised studies, the cluster-specific version of RoB 2 was applied. Non-randomised, quasi-experimental and pre–post-intervention studies were assessed using ROBINS-I [38]. Studies focused on protocols, intervention design or implementation processes without a primary causal effect estimate were not assessed with RoB 2 or ROBINS-I and were appraised using Kmet et al. [35] and considered narratively.

The risk-of-bias assessment was conducted at outcome level, focusing on the main outcome or set of outcomes included in the synthesis. The assessment was not used as an exclusion criterion, but rather to interpret the robustness of the evidence. Disagreements between reviewers were resolved by consensus and, when necessary, by consultation with a third reviewer (Supplementary Material S4).

### 2.5. Data Extraction

Data extraction was carried out in accordance with the PRISMA guidelines [19]. Two researchers independently collected relevant information from each study, including methodological characteristics, variables analysed, instruments used, and main results. In the event of discrepancies, a third researcher intervened to resolve them. The extracted data were organised into summary tables, which present the study characteristics and the variables assessed, respectively.

## 3. Results

### 3.1. Study Selection

The initial database search identified a total of 410 records. After removing 248 duplicate records, 162 records were screened based on their title and abstract. Of these, 102 records were excluded because they did not meet the eligibility criteria. Sixty full-text reports were assessed for eligibility, of which 47 were excluded. Thirteen studies from database searches were included. One additional study was identified by reviewing reference lists, and a further two studies were identified through searches for related articles in the databases. Consequently, 16 studies were included in the final synthesis (Figure 1).

### 3.2. Methodological Quality and Risk of Bias

The methodological quality scores assessed using Kmet et al. ranged from 0.69 to 0.91, indicating moderate to high methodological quality across the included studies (Table 1). The level of agreement between assessors was 0.876 (*p* < 0.001), indicating a good degree of agreement [36].

Overall, the studies demonstrated adequate methodological quality, although limitations were identified in some cases relating to sample size, the duration of interventions or the absence of standardised measures of implementation.

The supplementary assessment using the RoB 2 method revealed that several cluster-randomised trials presented ‘some concerns’ or ‘low risk’ overall, whilst two studies were considered to present a ‘high risk’ of bias [46,49]. The main concerns were related to incomplete reporting of allocation procedures, lack of blinding inherent to school-based interventions, missing outcome data, post-randomisation exclusions, use of self-reported behavioural outcomes and variability in intervention implementation.

The ROBINS-I assessment showed a higher risk of bias among non-randomised and quasi-experimental studies. Most studies were judged as having serious or critical risk of bias, mainly due to confounding, selection of participants, lack of random allocation, missing data, reliance on self-reported outcomes and limited availability of prespecified protocols. These findings were considered when interpreting the strength of the evidence, particularly for anthropometric outcomes and causal effectiveness claims.

Table 2 summarises the main methodological and design characteristics of the included studies. Overall, the studies analysed showed considerable heterogeneity in terms of methodological approaches, types of intervention and contexts of application. Furthermore, quasi-experimental quantitative studies or randomised controlled trials predominated, although research using mixed methodologies and studies focused on the evaluation of implementation processes were also identified [10,11,44].

All interventions took place in schools, at primary and secondary level, with participants aged between 6 and 18 years. Regarding the design of the interventions, a general trend was observed towards multi-component programmes combining nutrition education and the promotion of PA, sometimes incorporating behavioural, digital or family components [9,46,47]. The duration of the interventions varied, ranging from brief programmes lasting a few weeks to prolonged interventions lasting several months or even including follow-up. Given the substantial methodological and clinical heterogeneity among the included studies, a meta-analysis was not conducted. Consequently, the I^2^ statistic was not calculated, as the included studies did not provide sufficiently comparable effect estimates for the same outcomes, time points, populations and intervention contrasts.

Table 3 presents the variables analysed, the instruments used and the main results of the included studies, enabling the identification of the dimensions assessed in each intervention. In general, the studies addressed a combination of behavioural, cognitive and anthropometric variables, with frequent measurements relating to dietary habits, levels of PA and nutritional status [39,44,47]. Additionally, several studies incorporated cognitive and attitudinal variables, such as knowledge of nutrition or attitudes towards healthy lifestyles [41,45,50].

Furthermore, a significant group of studies focused on evaluating implementation, including aspects such as programme fidelity, acceptability, barriers and facilitators, as well as their sustainability within the school context [9,11]. This approach complements effectiveness analyses, providing key information on the feasibility and scalability of the interventions.

In terms of outcomes, most studies reported improvements in at least one of the dimensions assessed, particularly in eating habits, levels of PA or health-related knowledge [44,46,50]. However, the evidence on changes in anthropometric indicators, such as Body Mass Index (BMI), is more variable, suggesting a need for longer-term or more intensive interventions to achieve sustained effects [39,40].

Due to the heterogeneity in study design, intervention duration, age group, outcome measures and reporting of effect estimates, the findings were synthesised narratively. The synthesis was structured according to intervention duration. Shorter interventions (up to 12 weeks) [45,49,50] tended to report improvements mainly in knowledge, attitudes or selected behavioural outcomes, but provided limited evidence of anthropometric change. Medium-duration interventions (from 3 months to one school year) [39,40,41,42,43,44,47,51] more frequently reported changes in dietary behaviours, PA or sedentary behaviour, although effects on BMI and weight status were inconsistent. Longer interventions (one year or more than one year) [9,46,48] appeared more suitable for examining sustainability, school capacity and multilevel implementation processes, but their effects were strongly influenced by contextual and organisational factors.

Overall, the results showed that school-based interventions combining nutrition education and the promotion of PA tended to have positive effects, particularly on behavioural and cognitive variables. However, the lack of data on effect size or heterogeneity in study designs, measures used and dimensions assessed makes direct comparison between studies difficult. Furthermore, the inclusion of studies focusing on implementation highlighted the importance of contextual and organisational factors for the success of interventions, emphasising the role of the school environment, available resources and institutional support in the adoption and sustainability of programmes [9,10,11,49].

## 4. Discussion

The aim of this review was to analyse school-based programmes combining PA and nutrition, considering both their reported effects and the extent to which implementation-related outcomes were described. Based on the findings presented in the results, several notable elements can be identified in the interventions reviewed.

Firstly, it was observed that the studies analysed had a positive and significant impact in terms of PA or anthropometric measures [10,49,51], nutrition [41,42,50] or both variables [39,40,43,44,45,46,47,48], although the results were inconsistent in terms of the variables analysed. This aligns with and reinforces the idea put forward by previous research regarding the potential of PE and PA in the school context to improve habits related to these variables [3,4,6,14,17,61]. It therefore provides a relevant context for meaningfully addressing the rise in unhealthy habits highlighted by the WHO [1].

The studies also found improvements in community culture and capacity to promote healthy lifestyles [9,11,45,51]. These findings reinforce the conclusions of recent research highlighting the value of the educational context in promoting healthy lifestyles and increasing health literacy, the impact of which extends not only to pupils but also to their wider environment [2,5,6,8]. In this regard, studies indicating improvements that can be extrapolated to the community context or culture are directly linked to the competence of teachers and to teams of educational professionals who are aware of and committed to improving health and health literacy [12,13].

In the same way, several studies analysed the feasibility of programme implementation, sustainability, fidelity, and acceptability, as well as barriers and facilitators [9,11,45].

In this context, barriers such as the time required to implement the initiatives or the academic workload were identified, while institutional support, instructional design, community involvement, and a committed and well-prepared faculty were identified as key facilitators for these programmes [9,11,45]. These implementation factors are fundamental to the sustainability and success of interventions, which are closely related to the findings of previous studies [7,12].

On the other hand, studies that found improvements in anthropometric and/or PA-related aspects [10,49,51], those yielding positive data on nutrition or dietary habits [41,42,50], and those indicating improvements in both PA and nutrition [39,40,43,44,45,46,47,48] were consistent with the existing literature in terms of efficacy. In this regard, it can be noted that those studies that improved or aimed to improve variables related to PA, anthropometric measurements or sporting habits increased their effectiveness in longer-term programmes [15] and with greater contextual involvement [8,14]. With regard to programmes aimed at improving, or which did improve, dimensions related to nutrition or dietary habits require a more holistic approach [6,16], incorporating satisfaction with contextual and emotional [42] or cognitive [41,50] variables, with social and institutional support again being relevant to promote the success of the intervention [8,14]. This is consistent with previous reviews that focus on obesity prevention [18]. With regard to the duration of the interventions, it is worth noting that significant improvements were observed across a range of variables in all of them. Longer-term interventions [9,46,48], including those of medium duration [39,40,41,42,43,44,47,51], showed more profound significant changes in anthropometric measures, community culture, and nutritional and physical activity habits. In particular, the longer-term interventions [9,46,48] demonstrated greater sustainability, implementability and influence on the context. This is consistent with the previous literature regarding the greater effectiveness of longer-term programmes [15].

The findings presented in this systematic review should be interpreted with caution due to the limitations of the research. Although numerous studies report effects on health, dietary or PA variables, not all describe implementation-related aspects with sufficient precision. In many cases, information on fidelity, dose administered, reach, adoption, maintenance, costs or contextual barriers is limited or heterogeneous, making it difficult to draw direct comparisons between studies and to draw firm conclusions about the determinants of effectiveness. In addition, the assessment of the risk of bias in non-randomised studies indicated a risk of bias ranging from serious to critical. No minimum intervention dose or duration was established as an eligibility criterion. Although this allowed the review to capture the diversity of school-based programmes combining PA and nutrition, it also increased heterogeneity and limits the strength of cross-study comparisons. To address this issue, the narrative synthesis was structured according to intervention duration, study focus and outcome domain.

This lack of homogeneity reinforces the need to systematically incorporate implementation indicators into future research. In this regard, it should be noted that the study of implementation factors and the effectiveness of the studies was conducted in a general manner, incorporating aspects relating to BMI, screen time, frequency of PA, nutritional habits, and emotional or cognitive variables, amongst other issues. Consequently, the systematic review does not focus on a single variable, but rather addresses various factors present in the analysed studies. This provides a more comprehensive view of these variables but prevents a more concrete and specific focus on any one of them. For all these reasons, the results must be interpreted with caution, since the data contain such a wide variety of information that it is difficult to draw highly accurate conclusions. Similarly, a time limit of 6 years was established, which, whilst it may be seen as an advantage in terms of keeping the review up to date, may also be considered a limitation as it provides a narrower view of the current state of the field. Furthermore, the language aspect may be considered another limitation, as the languages of the reviewed studies were restricted to English, Spanish and Portuguese. Additionally, the lack of the grey literature or specialist journals may also be considered a limitation.

On the other hand, it should be noted that, although the educational and PE context offers numerous opportunities to promote health and health literacy, the potential of this context must be addressed through a holistic educational approach, rather than from a clinical perspective [62]. Consequently, future lines of research should analyse the various variables in a more specific manner and in relation to other psychological, social, socio-emotional and cognitive aspects, or those linked to the pupils’ areas of interest. About implementation factors, the findings suggested that future interventions should systematically integrate both effectiveness and implementation evaluation, to optimise their impact and facilitate their transfer to other educational contexts.

## 5. Conclusions

The results of this systematic review allow us to conclude that school programmes combining PA and nutrition education could be an effective strategy for health promotion in children and adolescents, especially when developed using multi-component, participatory approaches adapted to the educational context. Overall, the included studies show that this type of intervention can help improve knowledge, dietary habits, levels of PA, anthropometric indicators and other outcomes linked to child and adolescent well-being. However, the magnitude and stability of these effects appear to depend, to a large extent, on how the programmes are implemented in schools.

In relation to the main objective of the review, the findings highlight that the effectiveness of interventions cannot be interpreted solely on the basis of their final outcomes but must be analysed alongside the implementation factors that influence their delivery. Fidelity, acceptability, teacher training, availability of resources, involvement of the educational community, curricular integration and programme are emerging as key dimensions for facilitating the implementation of interventions that combine PA and nutrition. In this regard, studies that incorporate process evaluations, qualitative approaches or implementation frameworks such as RE-AIM provide particularly valuable information for identifying the mechanisms that facilitate or hinder the transfer of interventions into actual school practice.

Furthermore, the results suggest that the interventions with the greatest potential are those that do not merely convey information about healthy eating or PA, but rather modify the school environment, incorporate practical activities, involve teachers and families, and promote sustained changes in school routines. The inclusion of components such as improving school menus, promoting water consumption, reorganising healthy spaces, training school staff, and increasing real opportunities for PA appears to foster greater alignment between programme objectives and actual implementation conditions.

From an applied perspective, the results have significant implications for the design of education and public health policies. For school PA and nutrition programmes to be scalable and sustainable, it is not enough to demonstrate that they are effective under controlled conditions; it is necessary to ascertain whether they can be adopted by schools, whether they have sufficient resources, whether teachers receive adequate training, and whether the proposed activities can be realistically integrated into the school’s organisation. Thus, the review may be useful for opening new lines of research regarding the current state of the field. Similarly, the value of this systematic review in compiling the most recent experiences in PA and nutrition within the educational context should be highlighted. Consequently, it may be useful for teachers, school management teams and multidisciplinary teams committed to implementing PA and nutrition programmes in the educational context.

The available evidence supports the need to move towards more comprehensive interventions, better described and evaluated not only in terms of effectiveness, but also in terms of fidelity, acceptability, reach and sustainability. Explicitly incorporating implementation science into the design, evaluation and communication of these programmes will improve their practical utility and help to consolidate them as effective health promotion strategies in the school setting.

## Figures and Tables

**Table 1:** Quality assessment of the studies.

Studies	Observer 1	Observer 2	Average Score
Sanromán-Martínez et al. (2020) [39]	0.82	0.80	0.81
Barnes et al. (2021) [40]	0.89	0.85	0.87
Franceschi et al. (2021) [41]	0.71	0.80	0.75
Kwok et al. (2021) [42]	0.79	0.80	0.795
Šumonja & Jevtić (2021) [43]	0.75	0.80	0.77
van Dongen et al. (2022) [9]	0.86	0.85	0.855
Arellano-Gómez et al. (2023) [44]	0.82	0.82	0.82
Babic et al. (2023) [45]	0.75	0.80	0.77
Rawal et al. (2023) [46]	0.86	0.85	0.855
Rosenkranz et al. (2023) [10]	0.91	0.85	0.88
Gansterer et al. (2024) [47]	0.79	0.82	0.80
Kobel et al. (2024) [48]	0.88	0.85	0.86
Roman-Vinas et al. (2024) [49]	0.86	0.80	0.83
Chan et al. (2025) [11]	0.90	0.90	0.90
Gravino et al. (2025) [50]	0.68	0.70	0.69
Wang et al. (2026) [51]	0.92	0.90	0.91

**Table 2:** Main characteristics of the study sample.

Author(s)	Country	Participants	Age	Methodology	Design	Duration	Protocol	
							Control Group	Experimental Group
Sanromán-Martínez et al. (2020) [39]	Mexico	262 pupils from two full-time primary schools (131 control, 131 intervention)	6–12 years	Quantitative	Quasi-experimental, descriptive, analytical and longitudinal	4 months	‘Club de Leones’ primary school, with pre- and post-assessment without structured educational intervention	Survey on eating and exercise habits and anthropometric screening at the start and end. At the intervention school, 9 educational sessions for children (20 min each), 2 workshops for parents, 1 training workshop for teachers, implementation of daily PA (15 min Monday to Friday) and modification of the school menu with energy and nutritional adjustments were carried out. From the ‘Ricardo J. Zevada’ primary school, which underwent a comprehensive intervention involving nutrition education, PA and menu changes
Barnes et al. (2021) [40]	Australia	742 schoolchildren	9–12 years	Quantitative	Cluster-randomised controlled trial (cluster RCT, 2 × 2 factorial design)	9 months	Usual school practice (no intervention)	Group 1: PA intervention (150 min/week during school hours) Group 2: Nutritional intervention (improved packed lunch content) Group 3: Combined intervention (PA + nutrition)
Franceschi et al. (2021) [41]	Italy	478 primary school pupils	aged 6–11	Quantitative	Quasi-experimental	6–12 months	Control group receiving standard education	Digital educational intervention focused on nutrition and PA to improve knowledge of healthy habits
Kwok et al. (2021) [42]	China	553 secondary school students	13–17 years	Quantitative	Quasi-experimental	Twice a week (8 months) Once a week (6 weeks)	Consisting of a control group where the use of green spaces and satisfaction were measured	Two experimental groups. Group A undertook hydroponic gardening activities combined with health promotion activities (8 months) and Group B only participated in health promotion activities (6 weeks)
Šumonja & Jevtić (2021) [43]	Serbia	736 primary school pupils	NR	Quantitative	Experimental	1 school year (9 months)	Continued with their usual curriculum, without any specific intervention. They took part in surveys and assessment tests at the start and end of the school year	They undertook the Nutrition and PA Education Programme (NPAEP), based on integrated nutrition education, promotion of PA and practical workshops to encourage the consumption of healthy foods
van Dongen et al. (2022) [9]	The Netherlands	Secondary school students	NR	Mixed methods	Quasi-experimental	3 years	Four schools implemented the Dutch Healthy School approach, which is a common approach to healthy schools in The Netherlands	Four schools implemented the Fit Lifestyle at School and at Home (FLASH) programme to investigate the impact of community capacity on overweight levels through leadership, a participatory school culture, health promotion activities and local collaborative networks
Arellano-Gómez et al. (2023) [44]	Mexico	300 schoolchildren	6–12 years	Quantitative (participatory approach)	Quasi-experimental	6 months	School group with no intervention or with usual practice	Multi-component participatory intervention including nutrition education, promotion of PA, and involvement of the school and family community. Participatory school programme with structured nutrition education and PA promotion activities
Babic et al. (2023) [45]	Australia	89 primary school pupils (indigenous population)	8–12 years	Quantitative	Evaluation study (pre–post)	10 weeks	NR	‘Yantiin Kalabara—5 Ways to a Healthier You’ educational programme based on interactive workshops on healthy lifestyles
Rawal et al. (2023) [46]	India	1568 schoolchildren	Years 6 and 7	Quantitative	Quasi-experimental trial (likely school-based design; not strictly an RCT according to the title)	2 school years	Schools with standard curriculum without specific intervention	‘i-PROMISe plus’ educational intervention based on health literacy, which includes: Nutrition education (healthy eating habits) Promotion of PA Behavioural components (health literacy, decision-making) Structured school activities (educational sessions + materials)
Rosenkranz et al. (2023) [10]	USA	1766 primary school pupils	9–11 years	Mixed methods	Cluster-randomised trial	NR	Received the School Wellness Integration Targeting Child Health (SWITCH) programme in its standardised form	Received the School Wellness Integration Targeting Child Health (SWITCH) programme in an enhanced and individualised form. This consisted of support from the researchers and a bespoke plan for each school
Gansterer et al. (2024) [47]	Austria	157 primary school pupils	9–10 years	Quantitative	Intervention study (quasi-experimental/controlled)	6 months	Control group with usual practice (no structured digital intervention)	EDDY programme: web-based nutrition and PA intervention with email support
Kobel et al. (2024) [48]	Germany	1943 primary school pupils	7.1 ± 0.6 years	Quantitative	Randomised controlled trial	1 school year (9 months)	Received the standard school curriculum. Measurements were taken one year apart	Participated in the intervention using materials implemented by teachers, including 10–15 min active breaks and specific activities. The materials were provided to teachers. Families also received information to support intervention within the home environment
Roman-Vinas et al. (2024) [49]	Spain	281 primary school pupils	7.45 ± 0.34 years	Quantitative	Randomised controlled trial	3 months	Attended regular PE lessons	Received a 3-month integrated neuromuscular training intervention. This was incorporated into PE sessions during the warm-up. The intervention session lasted 20 min
Chan et al. (2025) [11]	Singapore	Primary school pupils	7–12 years	Mixed methods	Implementation study	NR	NR	Multi-component school-based intervention focusing on healthy diet and PA with implementation evaluation (fidelity, acceptability and context)
Gravino et al. (2025) [50]	Italy	60 secondary school students	14–18 years	Quantitative	Quasi-experimental	8–12 weeks	Control group receiving standard teaching	School-based educational intervention combining nutrition education and PA to prevent risky eating behaviours
Wang et al. (2026) [51]	China	1627 primary school pupils	8.5 years	Quantitative	Randomised controlled trial	1 year	Standard curricula	Pupils who were neither overweight nor obese received the OptiChild programme. Pupils who were overweight or obese received the SCIENT programme, which is based on PA and dietary guidance for families

NR: Not reported.

**Table 3:** Treatment variables and main outcomes.

Studies	Dimension Assessed	Variables	Instruments	Objective	Main Results	Effect Size of the Effect
Sanromán-Martínez et al. (2020) [39]	Overall effectiveness (nutrition + PA)	Anthropometric: BMI, nutritional status Behavioural: Dietary habits and PA Cognitive: Knowledge of healthy eating	Survey on eating and exercise habits based on a questionnaire previously validated in Chile and adapted from the study by Lera et al. [52]); anthropometric screening of weight, height and BMI in accordance with WHO [53] and Sentíes [54]; nutritional analysis of the school menu	To evaluate the effectiveness of an educational intervention on nutritional status, knowledge, dietary habits, PA and the implementation of healthy menus among schoolchildren aged 6 to 12	In the intervention group, normal weight increased from 49.5% to 71.7%; overweight decreased from 21.3% to 9.2% and obesity from 25.1% to 19%. There were significant differences in the frequency of food consumption and habits (*p* < 0.0001), with increased consumption of fruit, vegetables, water and healthy snacks, and reduced consumption of high-calorie foods. Sedentary behaviour also decreased and time spent on PA increased. There were no significant differences in average BMI between groups (*p* > 0.05)	NR
Barnes et al. (2021) [40]	Anthropometric effectiveness	Anthropometric: BMI, waist circumference Behavioural: Diet quality and PA	Objective anthropometric measurement (weight, height, BMI, waist circumference) Paediatric Quality of Life Questionnaire (PedsQ)	To assess the independent and combined efficacy of school-based PA and nutrition interventions on body weight status and quality of life in schoolchildren	The nutritional intervention increased the likelihood of being of normal weight The PA intervention reduced waist circumference No significant changes were observed in overall BMI There were no synergistic effects in the combined intervention No significant changes in quality of life	OR = 1.64 (95% CI: 1.07–2.50) for weight category (nutrition) Mean difference in waist circumference ≈ −1.86 cm (PA intervention)
Franceschi et al. (2021) [41]	Cognitive effectiveness	Cognitive: Knowledge of nutrition and PA Behavioural (potential): Healthy lifestyle habits (self-reported)	Internally validated ad hoc questionnaire (pre–post)	To evaluate the effectiveness of a digital intervention in improving health knowledge among vulnerable populations	Significant increase in nutritional knowledge Improved understanding of PA Greater awareness of healthy lifestyles	NR
Kwok et al. (2021) [42]	Behavioural and psychosocial	Behavioural: Use of green spaces, PA Psychosocial: Subjective well-being (happiness) Behavioural (diet): Dietary habits	Questionnaire to measure satisfaction with green spaces Delighted-Terrible Faces (DT-Faces) Scale [55] Global School-based Student Health Survey (GSHS) [56] Lickert scale on substance use Dietary habits over the last 30 days Hand hygiene over the last 30 days Emotions and feelings of friendship PA Patient Health Questionnaire-9 (PHQ-9) [57] Health-related Qo [58]	To investigate the effects of school hydroponic gardening integrated with health promotion activities on improvements in the use and management of green spaces; healthy lifestyle, mental health and health-related quality of life	The hydroponic gardening programme, integrated with health-promoting activities for pupils, was more effective and beneficial for the use and competence in green spaces. It also improved nutritional habits, as well as resistance to the consumption of harmful substances. The intervention groups showed higher levels of happiness compared to the control group. With regard to PA, no significant changes were observed between the experimental group and the control group	Competence and use of green spaces (*η*^2^ = 0.21) Distance to and use of green spaces (*η*^2^ = 0.09) Happiness (*η*^2^ = 0.03) Healthy habits (*η*^2^ = 0.03) Resistance to substance use (*η*^2^ = 0.03) Satisfaction with green spaces (*η*^2^ = 0.02) PA (*η*^2^ = 0.00819) Emotion and friendship (*η*^2^ = 0.00537) Depressive symptoms (*η*^2^ = 0.00310) Health-related quality of life (*η*^2^ = 0.00232) Hand hygiene (*η*^2^ = 0.00168)
Šumonja & Jevtić (2021) [43]	Anthropometric and behavioural effectiveness	Anthropometric: BMI Behavioural: Food intake and PA	Questionnaire on food and activities over the last 24 h Anthropometric measurements to calculate BMI	To assess the impact of a cross-curricular programme on nutrition and PA on food intake, PA and BMI among primary school pupils in grades 1 to 4 in Serbia	A significant increase in fruit consumption and a significant reduction in sedentary behaviour (television and video games) were achieved. Organised PA increased. Finally, BMI did not change significantly All of this would confirm that the programme implemented is useful for improving the nutritional and PA habits of primary school pupils	NR
van Dongen et al. (2022) [9]	Implementation (RE-AIM)	Implementation: Adoption, fidelity, maintenance (RE-AIM) Contextual: Organisational capacity, leadership, networks Behavioural: PA and diet	Interviews Diaries Meeting minutes Weight Height Waist circumference BMI	To assess the impact of the intervention on community capacity and capacity-building processes over a three-year period, and its effects on the BMI and waist circumference of secondary school students	Improvements were found in community capacity across all schools participating in the intervention. Specifically, improvements were observed in leadership, school sports culture and local collaborative networks, with the latter showing the most modest improvements	NR
Arellano-Gómez et al. (2023) [44]	Behavioural and anthropometric effectiveness	Behavioural: Eating habits, level of PA Cognitive: Health literacy Anthropometric: BMI, weight, height	Structured questionnaire on eating habits PA questionnaire for schoolchildren Standardised anthropometric measurement	To evaluate the effect of a participatory intervention on nutrition and PA in schoolchildren	Significant improvement in dietary patterns Increase in levels of PA Moderate improvement in health knowledge Tendency towards stabilisation/improvement in BMI	NR
Babic et al. (2023) [45]	Cognitive and behavioural effectiveness	Cognitive: Health knowledge Attitudinal: Attitudes towards healthy lifestyles Behavioural: Behaviours related to diet and PA	Ad hoc health and lifestyle questionnaire Structured pre–post-assessment	To evaluate the effectiveness of an educational programme among the indigenous school population	Significant improvement in health knowledge Positive changes in attitudes towards healthy habits Preliminary evidence of improved behaviours High cultural acceptability of the programme	NR
Rawal et al. (2023) [46]	Behavioural effectiveness (health literacy)	Behavioural: Diet, PA, sedentary lifestyle Cognitive: Health literacy	Ad hoc questionnaire adapted from validated instruments for adolescents, piloted in India; assesses diet, PA and sedentary behaviour	To assess the effect of an educational intervention based on health literacy on dietary, PA and sedentary behaviours in school-aged adolescents	Significant improvement in health literacy Improvement in dietary habits (increased consumption of healthy foods/reduction in unhealthy foods) Increase in levels of PA Positive behavioural changes associated with improved health literacy	NR
Rosenkranz et al. (2023) [10]	Implementation + effectiveness	Implementation: Support strategies, adherence, fidelity Behavioural: PA and well-being behaviours	Youth Activity Profile (YAP) [59]	To compare the effectiveness of the School Wellness Integration Targeting Child Health (SWITCH) intervention in a standard implementation and an enhanced, individualised implementation	There was a significant improvement in PA time and a reduction in screen time. However, there were no significant differences between the enhanced programme and the standard programme	NR
Gansterer et al. (2024) [47]	Behavioural and anthropometric effectiveness	Anthropometric: BMI z-score Behavioural: Dietary habits and level of PA	Objective anthropometric measurement (BMI z-score) Self-reported questionnaires on habits	To assess the impact of a digital intervention on body weight and health behaviours	Reduction in or stabilisation of the BMI z-score Positive changes in dietary behaviours Slight increase in PA	NR
Kobel et al. (2024) [48]	Behavioural effectiveness	Behavioural: Health behaviours (PA, diet) Anthropometric (possible): Health indicators	Height Weight BMI Questionnaire for families	To investigate whether the Join The Healthy Boat intervention had similar or differing effects on weight and health-related behaviour in children with high and low socioeconomic status	Following the intervention, no significant differences were found in terms of overweight status, PA or screen time. Statistically significant improvements were found among pupils from high socioeconomic backgrounds regarding skipping breakfast	Skipping breakfast (high socioeconomic status = OR = 5.34/*p* = 0.01)
Roman-Vinas et al. (2024) [49]	Physical effectiveness	Physical: Physical fitness Behavioural: PA Dietary: Adherence to the Mediterranean diet	Weighing scales Physical tests Accelerometers Kidmed [60] Sleep diaries Ad hoc questions on family studies	To assess changes in physical fitness following an integrated neuromuscular training intervention in primary school children To assess how lifestyle behaviours and parental education influence these changes	Following the intervention, improvements were observed in the running test. The girls showed improvements in the handgrip strength test, BMI and body fat percentage. Strength improved in participants with low PA levels or high socioeconomic status. Pupils from families with a lower educational level showed improvements in speed and agility, strength and cardiorespiratory fitness	Cohen’s *d* (*p* = 0.006) effect size indicates a greater effect in pupils from families with a low educational level in relation to speed and agility
Chan et al. (2025) [11]	Implementation (process)	Implementation: Fidelity, acceptability, barriers and facilitators Contextual: Organisational and school factors Behavioural (secondary): Diet and perceived PA	Programme evaluation questionnaires Semi-structured interviews Focus groups Implementation records	To analyse the implementation processes and theoretical mechanisms of a multi-component school-based intervention	High acceptance among teachers and pupils Identification of structural barriers (time, academic workload) Key facilitators: Institutional support and pedagogical design Behavioural theory facilitated adoption and sustainability	NR
Gravino et al. (2025) [50]	Cognitive and attitudinal effectiveness	Cognitive: Nutritional knowledge Attitudinal: Attitudes towards food Behavioural: PA and risky eating behaviours	Eating Attitudes Test (EAT-26) Nutrition knowledge questionnaire (ad hoc) PA questionnaire (ad hoc)	To assess the impact of a school-based intervention on the prevention of risky eating behaviours	Significant reduction in the risk of eating disorders Improved attitudes towards food Increased nutritional knowledge Moderate improvement in PA habits	NR
Wang et al. (2026) [51]	Multi-component effectiveness	Anthropometric: BMI Behavioural: Diet and PA Multilevel: School, family and clinical influences	Physical measurements	To evaluate a multilevel intervention targeting schools, families and clinical settings for the prevention of obesity in primary school children in China	Both interventions improved health behaviours. The OptiChild programme slowed the increase in BMI. The SCIENT intervention led to a decrease in BMI, resulting in a reduction in the prevalence of obesity from 24.8% to 18.9%	NR

NR: Not reported.

## Data Availability

No new data were created or analyzed in this study.

## Supplementary Materials

The following supporting information can be downloaded at: https://www.mdpi.com/article/10.3390/healthcare14132029/s1, SM S1: PRISMA 2020 Checklist [63]; SM S2: Examples of references excluded from the bibliography and cited in the text [15,22,23,24,25,26,27,28,29,30,31,32]; SM S3: PRISMA 2020 flow diagram; SM S4: risk of bias. RoB-2 and ROBINS-I analysis tables [9,10,39,40,41,42,43,44,45,46,47,48,49,50,51].

## Abbreviations

The following abbreviations are used in this manuscript:

WHO	World Health Organization
PA	Physical activity
PE	Physical education
PRISMA	Preferred Reporting Items for Systematic reviews and Meta-Analyses
PROSPERO	International prospective register of systematic reviews
BMI	Body Mass Index
NR	Not reported
NCG	No control group
NA	Not applicable
