# Supplementary material for: Assessment of the Implementation of Combined Physical Activity and Nutrition Programmes in Schools: A Systematic Review
Source: Healthcare (Basel). 2026 Jul 7;14(13):2029. doi: 10.3390/healthcare14132029 (PMC13360688; doi:10.3390/healthcare14132029)
Supplement: Supplementary file 1 [file healthcare-14-02029-s001.zip › SM S2. Examples of references excluded from the bibliography and cited in the text.pdf]

Examples of references excluded from the bibliography and cited in the text

1. Navidad, L.; Padial-Ruz, R.; González, M.C. Nutrition, Physical Activity, and New Technology Programs on Obesity Prevention in Primary Education: A Systematic Review. *Int. J. Environ. Res. Public Health* **2021**, *18*, doi:10.3390/ijerph181910187.
2. Al-Haroni, H.; Nik Farid, N.D.; Azanan, M.S. Effectiveness of Education Intervention, with Regards to Physical Activity Level and a Healthy Diet, among Middle Eastern Adolescents in Malaysia: A Study Protocol for a Randomized Control Trial, Based on a Health Belief Model. *PLoS One* **2024**, *19*, doi:10.1371/journal.pone.0289937.
3. Ashton, L.M.; Rayward, A.T.; Pollock, E.R.; Kennedy, S.-L.; Young, M.D.; Eather, N.; Barnes, A.T.; Lee, D.R.; Morgan, P.J. Twelve-Month Outcomes of a Community-Based, Father-Daughter Physical Activity Program Delivered by Trained Facilitators. *International journal of behavioral nutrition and physical activity* **2024**, *21*, 1–16, doi:10.1186/s12966-024-01648-w.
4. Al-Qahtani, A.M. Lifestyle Habits among Najran University Students, Najran, Saudi Arabia. *Front. Public Health* **2022**, *10*, doi:10.3389/fpubh.2022.938062.
5. Hoosen, F.; Faber, M.; Nel, J.H.; Steyn, N.P.; Senekal, M. Feasibility Testing of the Health4LIFE Weight Loss Intervention for Primary School Educators Living with Overweight/Obesity Employed at Public Schools in Low-Income Settings in Cape Town and South Africa: A Mixed Methods Study. *Nutrients* **2024**, *16*, doi:10.3390/nu16183062.
6. Pérez-Mármol, M.; Chacón-Cuberos, R.; García-Mármol, E.; Castro-Sanchez, M. Relationships among Physical Self-Concept, Physical Activity and Mediterranean Diet in Adolescents from the Province of Granada. *Children* **2021**, *8*, doi:10.3390/children8100901.
7. Michael, K.; Talias, M.A. Evaluation of a School Intervention to Improve Adherence to the Mediterranean Diet. *Health Educ. J.* **2024**, *83*, 215–227, doi:10.1177/00178969241232220.
8. Cepni, A.B.; Walsh, D.W.; Kim, H.; Yoon, C.Y.; Hughes, S.O.; Ledoux, T.A.; Johnston, C.A. Assessing the Efficacy of Sport-Based Physical Education on Children’s Activity Behaviors: A Randomized Controlled Trial. *J. Phys. Act. Health* **2025**, *22*, 205–215, doi:10.1123/jpah.2024-0243.
9. Mohammadi, S.; Su, T.T.; Papadaki, A.; Jalaludin, M.Y.; Dahlui, M.; Mohamed, M.N.A.; Jago, R.; Toumpakari, Z.; Johnson, L.; Abdul Majid, H.A. Perceptions of Eating Practices and Physical Activity among Malaysian Adolescents in Secondary Schools: A Qualitative Study with Multi-Stakeholders. *Public Health Nutr.* **2021**, *24*, 2273–2285, doi:10.1017/S1368980020002293.
10. O’Byrne, Y.; Dinneen, J.; Coppinger, T. Methodology for Evaluation of Complex School-Based Health Promotion Interventions. *J. Public Health Policy* **2024**, *45*, 623–638, doi:10.1057/s41271-024-00510-4.
11. Zhang, P.; Wang, Y.; Shen, F.; Xing, Y.-F.; Gu, J.; Li, X.-Y.; Jin, H.; Jin, S.-F.; Xu, M.; Wang, H.-J.; et al. Lifestyle Intervention in Children with Obesity and Nonalcoholic Fatty Liver Disease (NAFLD): Study Protocol for a Randomized Controlled Trial in Ningbo City (the SCIENT Study). *Trials* **2024**, *25*, doi:10.1186/s13063-024-08046-4.
12. Weaver, R.G.; Beets, M.W.; Adams, E.L.; Kaczynski, A.T.; Chen, B.; Armstrong, B.; Burkart, S.; Kiely, K.P.; Cepni, A.B.; White, J.W.; et al. Rationale and Design of Healthy Kids Beyond the Bell: A 2x2 Full Factorial Study Evaluating the Impact of Summer and after-School Programming on Children’s Body Mass Index and Health Behaviors. *Trials* **2024**, *25*, doi:10.1186/s13063-024-08555-2.
