# Supplementary material for: Assessment of the Implementation of Combined Physical Activity and Nutrition Programmes in Schools: A Systematic Review
Source: Healthcare (Basel). 2026 Jul 7;14(13):2029. doi: 10.3390/healthcare14132029 (PMC13360688; doi:10.3390/healthcare14132029)
Supplement: Supplementary file 1 [file healthcare-14-02029-s001.zip › SM S4. Risk of bias. RoB-2 and ROBINS-I analysis tables.pdf]

Risk of Bias Tables

Reviewer 1 assessment for inclusion as supplementary material

Table S1. RoB 2 assessment of randomised and cluster-randomised studies

Note. D1 = randomisation process; D1b = timing of identification/recruitment in cluster-randomised trials; D2 = deviations from intended interventions; D3 = missing outcome data; D4 = measurement of the outcome; D5 = selection of the reported result.

Study	RoB 2 version	Outcome assessed	D1	D1b	D2	D3	D4	D5	Overall judgement
Barnes et al. (2021) [40]	RoB 2 cluster-RCT	BMI, waist circumference, BMI category and quality of life at 9 months	Some concerns	Low risk	Some concerns	Some concerns	Some concerns	Some concerns	Some concerns
Kobel et al. (2024) [48]	RoB 2 cluster-RCT	Weight status and health-related behaviours after 12 months	Some concerns	Some concerns	Some concerns	Some concerns	Some concerns	Some concerns	Some concerns
Rawal et al. (2023) [46]	RoB 2 cluster-RCT	Diet and physical activity-related behaviours at two-year follow-up	Some concerns	Low risk	Some concerns	High risk	Some concerns	Some concerns	High risk
Roman-Viñas et al. (2024) [49]	RoB 2 cluster-RCT	Physical fitness and anthropometric outcomes after 3 months	Some concerns	Some concerns	High risk	Some concerns	Some concerns	Some concerns	High risk
Rosenkranz et al. (2023) [10]	RoB 2 cluster-RCT	Physical activity, sedentary screen time and dietary intake	Some concerns	Low risk	Some concerns	Some concerns	Some concerns	Some concerns	Some concerns
Wang et al. (2026) [51]	RoB 2 cluster-RCT	Change in BMI after one academic year	Some concerns	Low risk	Some concerns	Low risk	Low risk	Low risk	Some concerns

Table S2. ROBINS-I assessment of non-randomised and quasi-experimental studies

Note. D1 = confounding; D2 = selection of participants into the study; D3 = classification of interventions; D4 = deviations from intended interventions; D5 = missing data; D6 = measurement of outcomes; D7 = selection of the reported result.

Study	Outcome assessed	D1	D2	D3	D4	D5	D6	D7	Overall judgement
Sanromán-Martínez et al. (2020) [39]	Nutritional status, eating habits and physical activity	Serious	Moderate	Moderate	Moderate	Moderate	Serious	Moderate	Serious
Franceschi et al. (2021) [41]	Healthy lifestyle knowledge, dietary behaviour, PA and BMI z-score	Critical	Serious	Moderate	Moderate	Serious	Serious	Serious	Critical
Kwok et al. (2021) [42]	Dietary habits, green space use, lifestyle and wellbeing	Serious	Serious	Moderate	Moderate	Serious	Serious	Moderate	Serious
Šumonja & Jevtić (2021) [43]	Food intake, physical activity, sedentary time and BMI	Moderate	Moderate	Low/Moderate	Moderate	Moderate	Serious	Moderate	Serious
van Dongen et al. (2022) [9]	Community capacity, implementation processes, BMI and waist circumference	Serious	Serious	Moderate	Moderate	Moderate	Moderate/Serious	Moderate	Serious for effectiveness outcomes; Moderate for implementation/process evidence
Babic et al. (2023) [45]	Feasibility and preliminary changes in PA, nutrition and screen time	Critical	Serious	Moderate	Moderate	Moderate	Serious	Moderate	Critical for causal effectiveness; Moderate for feasibility/process interpretation
Gansterer et al. (2024) [47]	BMI z-score after 15-week web-based nutrition and PA intervention	Serious	Serious	Low/Moderate	Moderate	Serious	Low for BMI z-score	Moderate	Serious
Gravino et al. (2025) [50]	Eating habits, PA participation, self-esteem and emotional wellbeing	Critical	Serious	Moderate	Moderate	No information/Moderate	Serious	Serious	Critical

Additional note. Arellano-Gómez et al. (2023) and Chan et al. (2025) were not included in these tables because RoB 2 and ROBINS-I were not applicable to their primary study focus. These studies should be appraised using Kmet et al. and considered narratively as protocol/design or implementation/process evidence.

Risk of Bias Tables

Reviewer 2 assessment for inclusion as supplementary material

Table S3. RoB 2 assessment of randomised and cluster-randomised studies

Note. D1 = randomisation process; D1b = timing of identification/recruitment in cluster-randomised trials; D2 = deviations from intended interventions; D3 = missing outcome data; D4 = measurement of the outcome; D5 = selection of the reported result.

Study	RoB 2 version	Outcome assessed	D1	D1b	D2	D3	D4	D5	Overall judgement
Barnes et al. (2021) [40]	RoB 2 cluster-RCT	BMI, waist circumference, BMI category and quality of life at 9 months	Low risk	Low risk	Low risk	Low risk	Low risk	Low risk	Low risk
Kobel et al. (2024) [48]	RoB 2 cluster-RCT	Weight status and health-related behaviours after 12 months	Low risk	Low risk	Some concerns	Some concerns	Some concerns	Low risk	Some concerns
Rawal et al. (2023) [46]	RoB 2 cluster-RCT	Diet and physical activity-related behaviours at two-year follow-up	Some concerns	Some concerns	Low risk	High risk	Some concerns	Some concerns	High risk
Roman-Viñas et al. (2024) [49]	RoB 2 cluster-RCT	Physical fitness and anthropometric outcomes after 3 months	Some concerns	Some concerns	High risk	Low risk	Some concerns	Low risk	High risk
Rosenkranz et al. (2023) [10]	RoB 2 cluster-RCT	Physical activity, sedentary screen time and dietary intake	Low risk	Low risk	Low risk	Some concerns	Some concerns	Low risk	Some concerns
Wang et al. (2026) [51]	RoB 2 cluster-RCT	Change in BMI after one academic year	Low risk	Low risk	Low risk	Low risk	Low risk	Low risk	Low risk

Table S4. ROBINS-I assessment of non-randomised and quasi-experimental studies

Note. D1 = confounding; D2 = selection of participants into the study; D3 = classification of interventions; D4 = deviations from intended interventions; D5 = missing data; D6 = measurement of outcomes; D7 = selection of the reported result.

Study	Outcome assessed	D1	D2	D3	D4	D5	D6	D7	Overall judgement
Sanromán-Martínez et al. (2020) [39]	Nutritional status, eating habits and physical activity	Serious	Moderate	Low	Moderate	No information	Serious	Moderate	Serious
Franceschi et al. (2021) [41]	Healthy lifestyle knowledge, dietary behaviour, PA and BMI z-score	Serious	Moderate	Moderate	Serious	Serious	Serious	Moderate	Serious
Kwok et al. (2021) [42]	Dietary habits, green space use, lifestyle and wellbeing	Serious	Serious	Moderate	Serious	Serious	Serious	Moderate	Serious
Šumonja & Jevtić (2021) [43]	Food intake, physical activity, sedentary time and BMI	Moderate	Moderate	Low	Moderate	Moderate	Serious	Moderate	Serious
van Dongen et al. (2022) [9]	Community capacity, implementation processes, BMI and waist circumference	Serious	Serious	Moderate	Serious	Moderate	Moderate	Moderate	Serious
Babic et al. (2023) [45]	Feasibility and preliminary changes in PA, nutrition and screen time	Serious	Moderate	Low	Low	Moderate	Serious	Moderate	Serious
Gansterer et al. (2024) [47]	BMI z-score after 15-week web-based nutrition and PA intervention	Serious	Serious	Low	Moderate	Serious	Moderate	Moderate	Serious
Gravino et al. (2025) [50]	Eating habits, PA participation, self-esteem and emotional wellbeing	Serious	Moderado	Low	Moderate	No information	Serious	Moderate	Serious

Additional note. Arellano-Gómez et al. (2023) and Chan et al. (2025) were not included in these tables because RoB 2 and ROBINS-I were not applicable to their primary study focus. These studies should be appraised using Kmet et al. and considered narratively as protocol/design or implementation/process evidence.
