# Supplementary figures and images for: Assessment of the Implementation of Combined Physical Activity and Nutrition Programmes in Schools: A Systematic Review
Source: Healthcare (Basel). 2026 Jul 7;14(13):2029. doi: 10.3390/healthcare14132029 (PMC13360688; doi:10.3390/healthcare14132029)

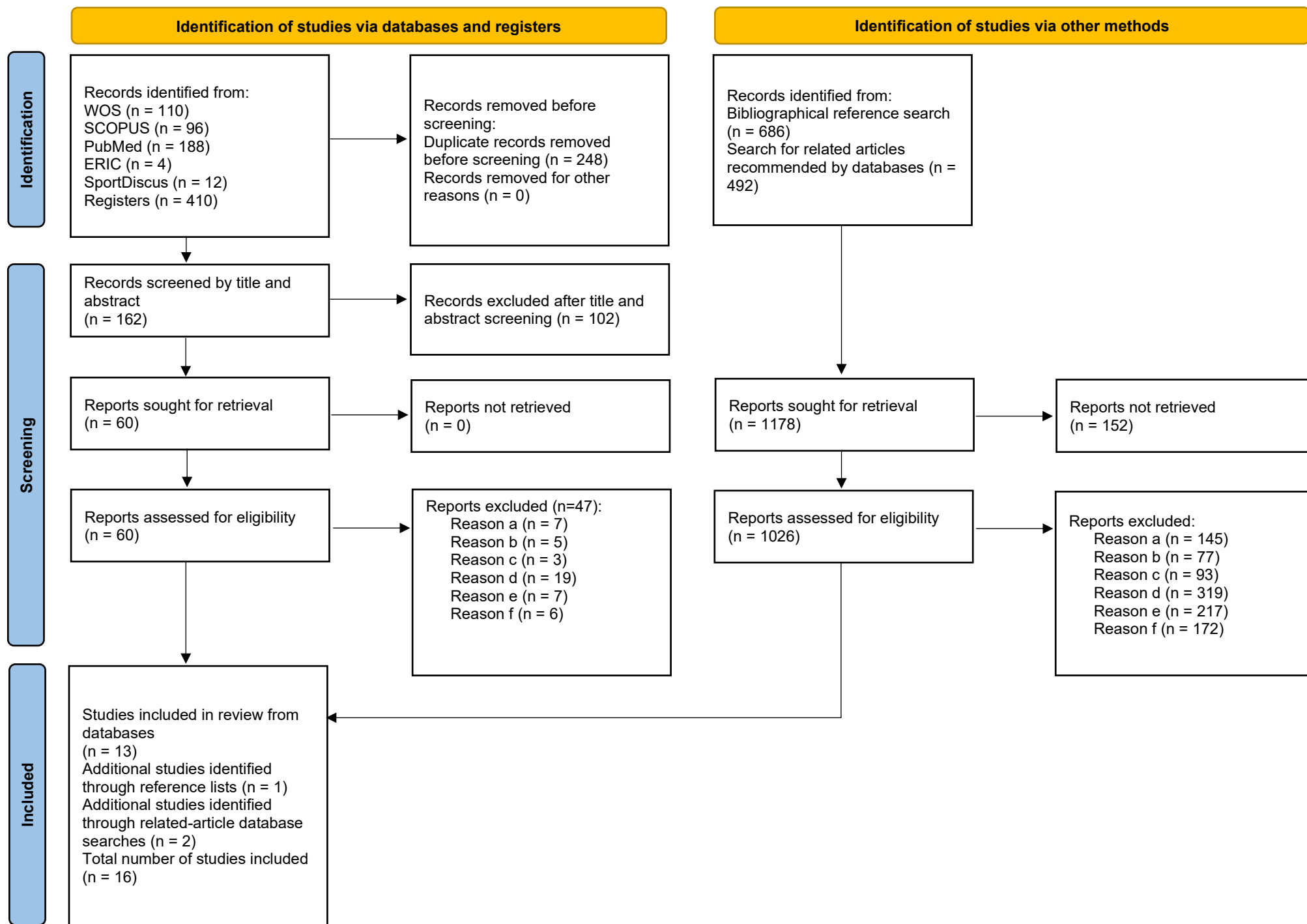

Supplement: Supplementary file 1 [file healthcare-14-02029-s001.zip › SM S3. PRISMA 2020 flow diagram.pdf]
